# Human α-synuclein overexpression in a mouse model of Parkinson’s disease leads to vascular pathology, blood brain barrier leakage and pericyte activation

**DOI:** 10.1038/s41598-020-80889-8

**Published:** 2021-01-13

**Authors:** Osama Elabi, Abderahim Gaceb, Robert Carlsson, Thomas Padel, Rana Soylu-Kucharz, Irene Cortijo, Wen Li, Jia-Yi Li, Gesine Paul

**Affiliations:** 1 Translational Neurology Group, Department of Clinical Science, Wallenberg Neuroscience Center and Wallenberg Center for Molecular Medicine, Lund University, Sölvegatan 17, 22184 Lund, Sweden; 2 Brain Disease Biomarker Unit, Department of Experimental Medical Science, Wallenberg Neuroscience Center, Lund University, 22184 Lund, Sweden; 3 Neural Plasticity and Repair Unit, Department of Experimental Medical Science, Wallenberg Neuroscience Center, Lund University, 22184 Lund, Sweden; 4 Institute of Health Sciences, China Medical University, Shenyang, 110122 China; 5 Department of Neurology, Scania University Hospital, 22185 Lund, Sweden

**Keywords:** Neuroscience, Physiology, Neurological disorders

## Abstract

The pathological hallmark of Parkinson’s disease (PD) is the formation of Lewy bodies containing aggregated alpha-synuclein (α-syn). Although PD is associated with these distinct histological changes, other pathological features such as microvascular alterations have been linked to neurodegeneration. These changes need to be investigated as they create a hostile brain microenvironment and may contribute to the development and progression of the disease. We use a human α-syn overexpression mouse model that recapitulates some of the pathological features of PD in terms of progressive aggregation of human α-syn, impaired striatal dopamine fiber density, and an age-dependent motor deficit consistent with an impaired dopamine release. We demonstrate for the first time in this model a compromised blood–brain barrier integrity and dynamic changes in vessel morphology from angiogenesis at earlier stages to vascular regression at later stages. The vascular alterations are accompanied by a pathological activation of pericytes already at an early stage without changing overall pericyte density. Our data support and further extend the occurrence of vascular pathology as an important pathophysiological aspect in PD. The model used provides a powerful tool to investigate disease-modifying factors in PD in a temporal sequence that might guide the development of new treatments.

## Introduction

Parkinson's disease (PD) is the second most common neurodegenerative disorder and pathologically characterized by the progressive degeneration of the nigrostriatal system resulting in rigidity, bradykinesia and resting tremor^1^. The most affected cells are dopaminergic (DA) neurons in the substantia nigra pars compacta (SNpc). The pathological hallmark of PD is the formation of Lewy bodies containing aggregated alpha-synuclein (α-syn)^2^. Although PD is associated with these distinct histological changes, concomitant pathological alterations are gaining importance as they might sustain or aggravate the neuronal degeneration.

Disruption of the blood–brain barrier (BBB) and other microvascular alterations are increasingly recognized as a common denominator of several neurodegenerative diseases such as Alzheimer’s disease (AD) and Huntington’s disease^3^. Also in PD, post-mortem and cerebrospinal fluid (CSF) analyses have indicated changes in small blood vessels and BBB leakage as part of the pathology^4–10^.

A key player in maintaining vascular function is the pericyte, a perivascular cell that is uniquely positioned at the blood/brain interface^11^. Data is emerging that pericyte changes occur in certain neurological disorders and are associated with or even precede neuronal loss^12–14^. This is important as pericytes not only maintain the anatomical, biochemical and immune functions of the BBB, they also regulate capillary flow, angiogenesis and the clearance of toxic products^15,16^. One of the responses of pericytes to pathology is their activation which is characterized by expression of markers such as regulator of G-protein signaling 5 (RGS5) and neuron-glial antigen 2 (NG2)^17–19^, and activation of pericytes has been observed in neurodegenerative disorders^12,20^.

In order to develop new therapies, it is essential to understand the underlying pathological processes that might aggravate the neurodegeneration in PD. This requires disease models that recapitulate such pathological changes. Toxin-induced PD animal models using e.g. neurotoxins such as 1-methyl-4-phenyl-1,2,3,6-tetrahydropyridine (MPTP) or 6-hydroxydopamine (6-OHDA) either achieve a rapid loss of nigrostriatal DA neurons or a slower degeneration, depending on the dose regime or location of the toxin applied^21^. These neurotoxins alter mitochondrial function in DA neurons and increase oxidative stress and neuroinflammation^21,22^. Toxin-induced models are invaluable in studying certain aspects of the disease and some of the findings on vascular damage and BBB leakage have been shown in these models^23–25^, however, these models rarely form α-syn aggregates^26–28^, and only partially reflect the age-dependent slowly progressive nature of the PD pathology^29^.

Different PD models have been created overexpressing wildtype or mutated forms of α-syn by either genetic modification of animals or viral vector injection or by intracerebral administration of different forms of α-syn, such as monomers, oligomers and fibrils^30,31^. Interestingly, these models develop aggregated α-syn and/or α-syn-mediated neuronal loss, defining pathological hallmarks of PD^30^ and are therefore well positioned to understand the pathogenic mechanisms underpinning PD. Vascular changes, however, have not yet been examined in α-syn PD models.

Here we use a transgenic PD mouse model where the human wildtype α-syn gene is fused to the Green fluorescent protein (GFP) gene and overexpressed under the mouse α-syn promoter using the bacterial artificial chromosome (BAC)-α-syn-GFP construct^32^. This mouse model has previously been characterized and shown to exhibit an age-dependent accumulation of phosphorylated (p)-α-syn, behavioural impairments and dopamine system abnormalities of PD^32^. Here, we confirm and further characterize the progressive neuropathology of the model and examine whether stage-related microvascular changes are present. In particular, we investigate BBB leakage, changes in vessel and pericyte density, changes in collagen IV (Col IV) expression and pericyte activation at three different ages.

## Materials and methods

### Animals

In the present study, we used C57BL/6J male mice (n = 66) aged 3, 8 and 13 months reflecting different ages and stages of pathology. Mice are either referred to as wildtype (WT) mice (n = 26) or transgenic homozygote mice (TG) (n = 40). Numbers of animals for individual analysis are specified in the figure legends.

In TG mice, the human wildtype α-syn gene is fused to the GFP gene and overexpressed under the mouse α-syn promoter using the BAC-α-syn-GFP construct^32^. In these mice, α-syn-GFP is expressed in multiple brain regions, including the SNpc and the striatum as previously described^32^. Animals were housed in a 12 h (h) light/dark cycle with free access to food and water. All experimental procedures were carried out in accordance with the European Directive 2010/63/EU guidelines and approved by the Ethical Committee at Lund University.

### Behavioural tests

To assess motor performance in mice, open field and rotarod tests were performed at the age of 3, 8 and 13 months. For all tests, animals were habituated to the room at least 2 h before the test. For the open field test, animals were placed in an open field arena for 1 h in the dark and the general locomotor activity was evaluated recording the total distance using a behavioural tracking software (ANY-maze). Several days later, an amphetamine-induced open field test was performed. Mice were injected intraperitoneally (i.p) with amphetamine (5 mg/kg diluted in 0.9% sterile saline) and the locomotor activity was recorded in the same way as above.

For the rotarod test, animals were placed on a rod turning with increasing speed from 4 to 40 rpm in 5 min using Rotamex 4/8 (Columbus instruments). All animals were habituated to the instrument by performing a trial test. Three additional tests were performed spaced by at least 15 min. The latency to fall from the rod was recorded and the mean of the last 3 trials was calculated.

### Western blot and fractionation of α-syn

One brain hemisphere per mouse was cut to small pieces on ice. Fractionation and lysis were done as previously described^33^, with four washing steps of the 1% Triton X-100 insoluble pellet in 1 ml lysis buffer. Protein concentrations were measured with BCA kit using standard procedures (Thermo scientific, Pierce BCA protein kit, cat no #23225). Western blots were run using standard methods with a maximum of 14 μg protein per well. Antibodies used for the detection of α-syn were mouse anti-α-syn-(BD #610786, 1:1000) and rabbit anti-pS129-α-syn (Abcam #ab51253, 1:1000). Species-specific proteins were distinguished by molecular weight. Images were acquired in Imagelab (Chemidoc MP) version 5.2.1 and quantified in Fiji-ImageJ. Methods are described in detail in the Supplementary Information.

### Tissue processing

For immunohistochemistry, mice at the age of 3, 8 and 13 months were deeply anesthetized with sodium pentobarbital (Apoteksbolaget, Sweden) and transcardially perfused with 0.9% saline for 3 min followed by 4% paraformaldehyde (PFA) for 5 min. Brains were collected, kept in 4% PFA solution for 4 h, stored in a 25% sucrose solution for several days and then cut coronally at 30 µm thick sections.

### Immunohistochemistry

For immunohistochemical staining, sections were quenched with a peroxidase solution (3% H_2_O_2_, 10% methanol, diluted in phosphate buffer saline (PBS)) for 10 min before blocking for 1 h at room temperature (RT) in 3% serum diluted with 0.25% TritonX-100- PBS (PBS-TX) (Alfa Aesar). Primary antibodies diluted with 1% serum in PBS-TX were incubated overnight (O/N) at RT for rabbit anti-tyrosine hydroxylase (TH; 1:1000, Millipore) and rat anti-CD11b (1:200, Serotec) or 2 h at RT followed by O/N incubation at 4 °C for rat anti-dopamine transporter (DAT, 1:1000, Millipore). Sections were then incubated for 2 h at RT with the corresponding biotinylated secondary antibodies (1:200, Vector Laboratories), followed by 1 h incubation with an avidin–biotin kit (Vectastain Elite ABC kit, Vector Laboratories) and the staining was revealed using chromogen 3,3-diaminobenzidine (DAB, Peroxidase Substrate Kit, Vector Laboratories).

For double immunofluorescence staining, sections were washed 3 times for 10 min with PBS. The sections were blocked with 5% serum in PBS-TX for 1 h at RT and incubated with the following primary antibodies diluted with 3% serum in PBS-TX O/N at RT: goat anti-podocalyxin (PDCLX; 1:200, R&D Systems); rat anti-CD13 (1:200, Bio-Rad); rabbit anti-fibrinogen (1:400, Abcam), rabbit anti-NG2 (1:200, Millipore), anti-p-α-syn antibody (pS129; 1:500; Abcam); rabbit anti- Col IV (1:500, Bio-Rad) or rabbit anti-platelet-derived growth factor receptor β (PDGFRβ; 1:200, Cell Signalling). For PDGFRβ and p-α-syn staining, antigen retrieval was performed where sections were incubated in citrate buffer (PH = 6) for 30 min at 80 °C. For p-α-syn staining, the primary antibody was incubated with 10% horse serum and 0.1% bovine serum in PBS-TX O/N at 4 °C.

Sections were then incubated for 1 h at RT with the respective fluorophore-tagged secondary antibodies: CY3-conjugated donkey anti-rat IgG or Alexa fluor 647 donkey anti-rabbit diluted with 3% serum in PBS-TX (1:500; Jackson ImmunoResearch) or with anti-goat biotinylated secondary antibody (1:200, Vector Laboratories) followed by fluorophore-conjugated streptavidin Alexa fluor 647 (1:500; Jackson ImmunoResearch). Sections were mounted on gelatinised slides and coverslipped with mounting medium (PVA/DABCO).

### Confocal microscopy and image analysis

Confocal images were sampled from striatal sections according to AP + 0.62 to + 1.18 relative to bregma^34^ using a Nikon A1 or LEICA DMi8 confocal microscope. The collected images were obtained from a z-stack size of 10 µm at a step size of 1 µm unless specified differently in the figure legends. For maximum image projection, ImageJ software (NIH, USA) was used for z-stack image reconstruction. The same acquisition settings were applied for each image. To analyse α-syn and p-α-syn in endothelial cells or pericytes, orthogonal images were obtained to visualize a possible colocalization of α-syn-GFP and p-α-syn with either PDCLX or CD31 staining for endothelial cells, and PDGFRβ or CD13 staining for pericytes.

### α-Synuclein quantification

α-syn-GFP and pS129-α-syn fluorescence density was measured using ImageJ (NIH, USA). Z-stack images at 63 × magnification were obtained from the dorsal striatum and reconstructed for the maximum projection (z-stack size of 4 µm at a step size of 0.3 µm). The fluorescence density was calculated as the total fluorescence density subtracted from the background density.

### Blood vessel and pericyte density

Striatal blood vessel (PDCLX, Col IV) and pericyte (CD13) density were analyzed using the area fraction measurement tool of ImageJ software (NIH, USA). The density was expressed as the percentage of the PDCLX^+^, CD13^+^ area or Col IV^+^ area. The relationship between Col IV and PDCLX was expressed as ratio by calculating the density of Col IV divided by the density of PDCLX.

### Coverage of activated pericytes

The coverage of activated pericytes was assessed by calculating the proportion of NG2^+^ pericytes covering PDCLX^+^ vessels. The colocalization plugin tool of ImageJ (NIH, USA) was used to define and highlight the colocalized points NG2^+^ PDCLX^+^ in the z-stack image. The images were reconstructed for a maximum projection and the NG2^+^ PDCLX^+^ colocalized area was measured and expressed as a percentage of the total PDCLX^+^ area per image. Parenchymal NG2-staining that did not colocalize with PDCLX^+^ vessels was excluded from the analysis.

### Extravascular fibrinogen quantification

To quantify vascular leakage, extravascular fibrinogen was analyzed. PDCLX-staining was used to delineate vessels and to identify and exclude intravascular fibrinogen. The area covered by extravascular fibrinogen was analyzed and reported as the percentage of the total area analyzed using ImageJ (NIH, USA).

### DA neurons and fiber quantification

DA neurons labelled with TH were counted in the SNpc at 10 × magnification using the Olympus BX53 microscope and CellSens software. TH^+^ cell numbers were assessed at the level where the SNpc could be delineated from the ventral tegmental area by the medial terminal accessory nucleus of the optic tract (approx. − 3.16 relative to bregma)^34^. The density of TH^+^ and DAT^+^ fibres was evaluated in striatal sections (AP + 0.62 to + 1.18 relative to bregma)^34^. Images were obtained at 4 × magnification (Olympus BX53 microscope) and analysed by ImageJ (NIH, USA). The optical density was normalized to the cortex.

### Microglia quantification

Microglial cells labelled with CD11b were counted in striatal sections (AP + 0.62 to + 1.18 relative to bregma)^34^ at 20 × magnification using the Olympus BX53 microscope and CellSens software. Three to 4 sections per animal were analysed.

### Statistical analysis

Statistical analysis was performed using IBM SPSS version 20 software unless otherwise stated. Data are shown as mean ± standard error of the mean (SEM). Data were assessed for normal distribution based on Q–Q plot and considered independent. To assess statistical significance for normally distributed data, we used the parametric unpaired Student’s t-tests for comparisons between the two groups and considered significance at a p-value < 0.05. For comparison between multiple groups we used a one-way ANOVA followed by Tukey’s multiple comparison correction and significance was set at a p-value < 0.05. For comparison between multiple groups and treatments, data was analyzed using a two-way ANOVA followed by Tukey’s multiple comparison test and significance considered at a p-value < 0.05.

## Results

### Progressive accumulation and aggregation of α-syn

**Figure 1:**
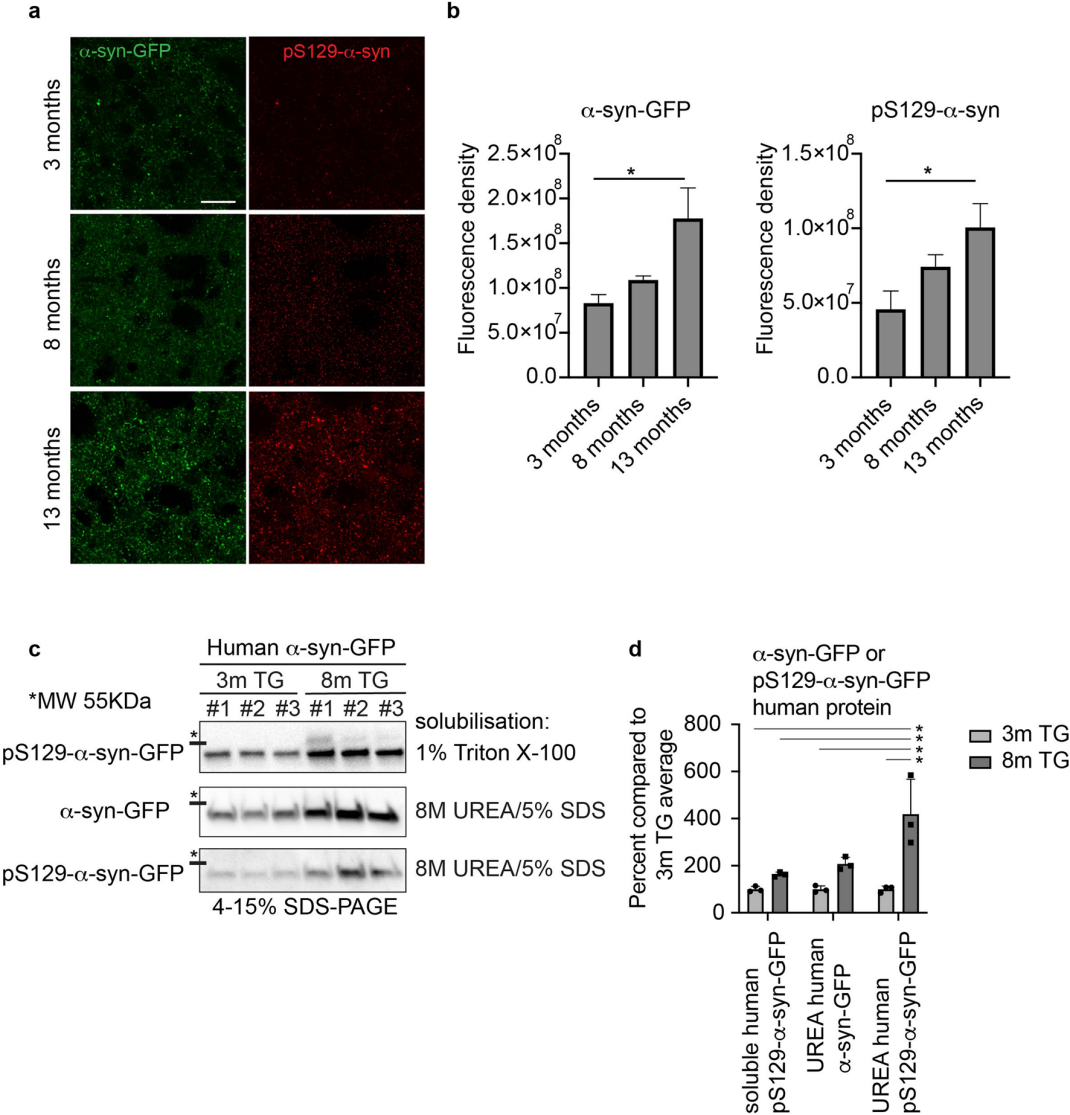
Progressive accumulation and aggregation of α-syn and p-α-syn in the striatum of the TG mice. (**a**) Reconstructed confocal image showing the fluorescence density level of *α*-syn-GFP and pS129-*α*-syn in the dorsolateral striatum of TG mice at age 3, 8, and 13 months (Z thickness 4 μm, step size 0.3 μm). Scale bar: 20 μm. (**b**) Corresponding quantification of the *α*-syn-GFP and pS129-*α*-syn fluorescence density in the dorsolateral striatum of TG mice at age of 3 months (n = 3), 8 months (n = 3) and 13 months (n = 3). One-way ANOVA: *p-value < 0.05. (**c**) Fractionation of 1% Triton-X-100 soluble, and insoluble *α*-syn solubilized in 8 M urea, 5% SDS. Human *α*-syn-GFP and human pS129-*α*-syn-GFP protein was quantified in Image J from western blots based on the mobility compared to the mouse *α*-syn. Images from the western blots of the Triton X-100 soluble human *α*-syn compared to human *α*-syn and pS129-*α*-syn in the Triton X-100 insoluble fraction. The full-length blots are presented in Supplementary Fig. 1c. Data in (**c**) were quantified in (**d**), two way-ANOVA, Tukey multiple correction analysis: *p-value < 0.05. Human *α*-syn was quantified by western blot in the Triton X-100 soluble, and wash 4 fractions (Supplementary Fig. 1b). α-syn-GFP = alpha-synuclein green fluorescent protein, pS129-α-syn = phospho S129-alpha-synuclein.

First, we investigated the accumulation and aggregation of human α-syn in this model. We showed a significant increase in the fluorescence density of α-syn-GFP and of p-α-syn as an indicator of aggregated α-syn in the striatum of TG mice between 3 and 13 months indicating a progressive accumulation and aggregation of human α-syn (α-syn-GFP, p = 0.041; p-α-syn, p = 0.049) (Fig. 1a,b)^32^. There was a trend that did not reach significance in the fluorescence density of these markers between age 3 and 8 months.

In order to further validate that the overexpression of human α-syn-GFP fusion protein in this model indeed results in a progressive aggregation of α-syn-GFP, we measured soluble versus insoluble human α-syn by western blotting (Fig. 1c,d). We found, that human-pS129-syn can be detected at 3 months but is not enriched in the insoluble fraction. However, at 8 months there was significantly more human pS129-syn in the insoluble fraction than in the insoluble fraction at 3 month, showing that human pS129-syn aggregates in TG mice in an age-dependent manner (p = 0.034) (Fig. 1c,d). Control experiments for total protein concentration showed very low levels of human α-syn-GFP in the supernatant from the last wash of the insoluble fraction pellet indicating that the fractionation was successful (Supplementary Fig. S1).

The accumulation of human α-syn in the striatum was followed by a progressive increase in the number of CD11b^+^ cells. Quantification of CD11b^+^ microglia showed an age-related increase of the number of CD11b^+^ cells in both WT and TG mice that was significantly higher in TG mice at all ages compared to age-matched controls and showed the morphology of activated microglia in TG mice at 13 months of age (Supplementary Fig. S2).

### Absence of nigral TH^+^ cell loss but significantly reduced TH^+^ and DAT^+^ fiber density in TG mice

**Figure 2:**
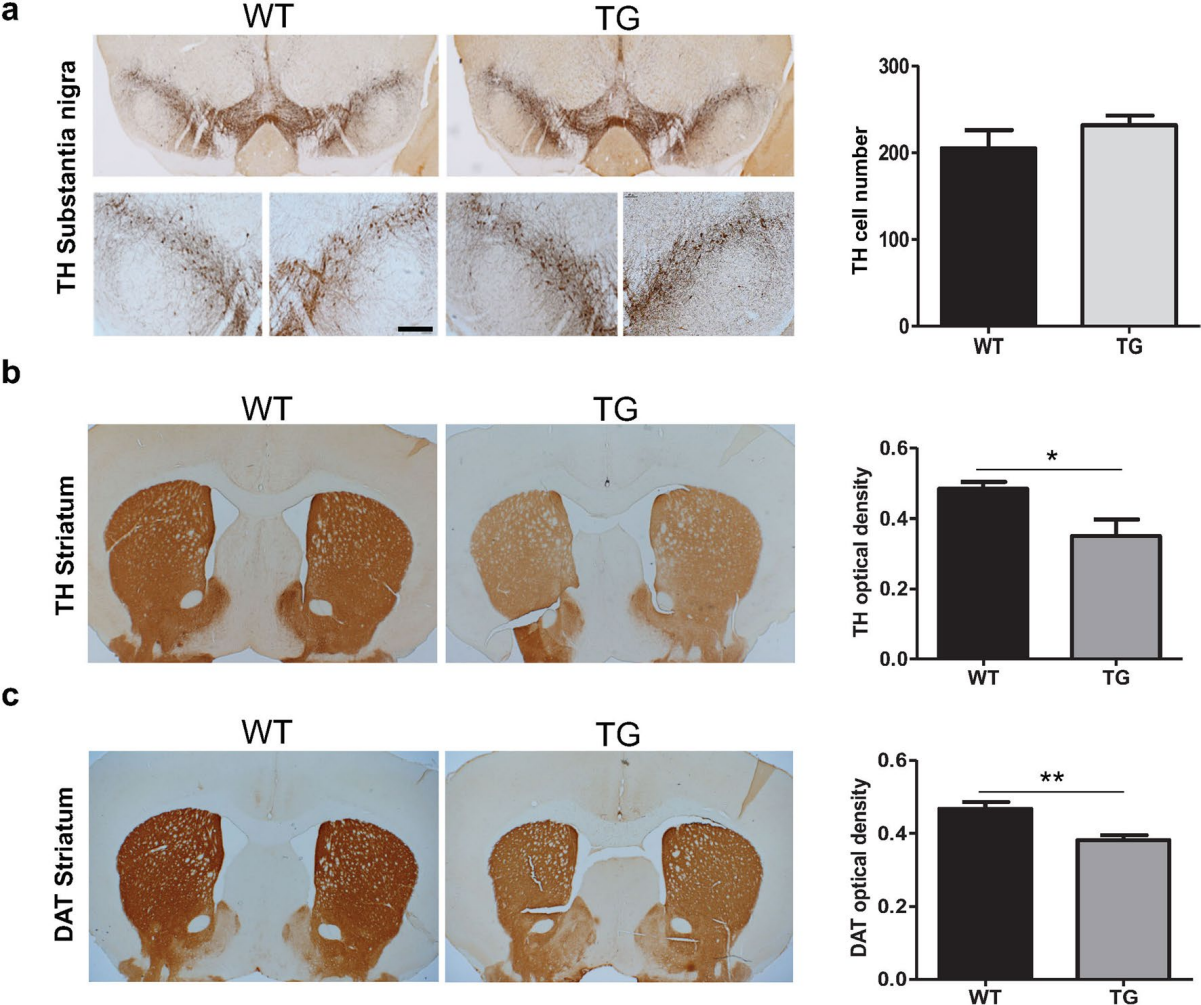
TH cell survival and reduction in TH and DAT density in TG mice. (**a**) TH^+^ staining and TH^+^ cell count in the SNpc of TG and WT mice at age 13 months (n = 4 WT, 5 TG). (**b**) TH^+^ fibre optical density in the striatum of TG and WT mice at age 13 months (n = 5 WT, 5 TG). (**c**) DAT^+^ fibre optical density in the striatum of the TG and WT mice at age 13 months (n = 3 WT, 5 TG). Two-tailed student’s t-test: *p-value < 0.05, **p < 0.01. Scale bar: 50 μm. DAT = Dopamine transporter, TH = Tyrosine hydroxylase.

We next investigated the impact of α-syn overexpression on the nigrostriatal DA system at 13 months. We assessed the number of TH^**+**^ neurons in the SNpc in TG and age-matched WT mice. The total number of TH^**+**^ labeled DA neurons in the SNpc was not different between TG and WT mice (Fig. 2a), consistent with previous findings in this model^32^. However, we observed a significant reduction in the density of striatal TH^**+**^ fibers and DAT^+^ fibers in the TG mice compared to WT mice (TH^**+**^ fibers, p = 0.03; DAT^**+**^ fibers, p = 0.008) (Fig. 2b,c).

### Age-dependent behavioural impairment in TG mice

**Figure 3:**
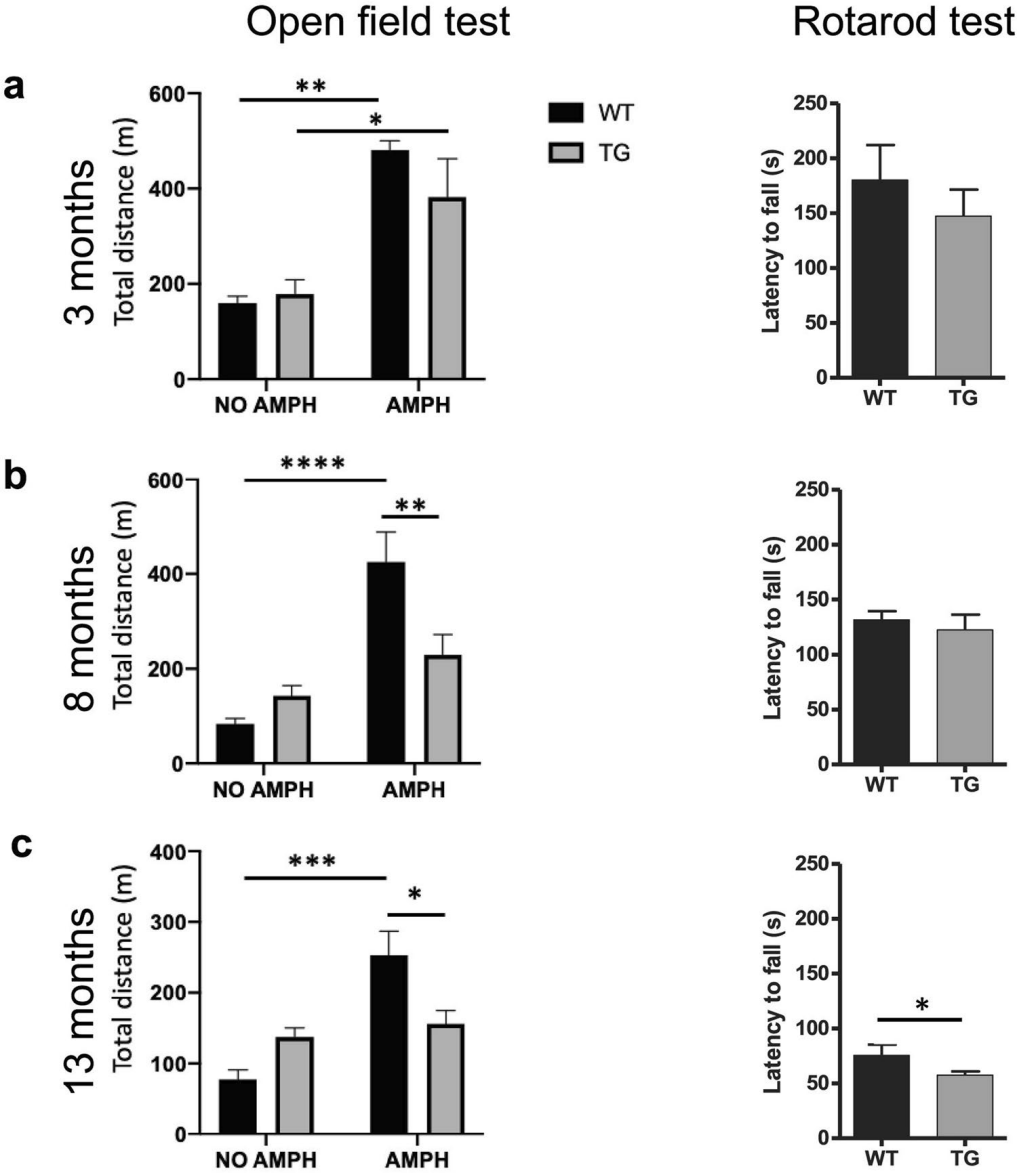
Progressively impaired motor function in TG mice. Open field motor test (left panel) showing distance travelled by WT and TG mice at naive condition and after amphetamine injection (5 mg/ kg) at (**a**) age of 3, (**b**) age of 8 and (**c**) age of 13 months, two-way ANOVA: *p-value < 0.05, **p < 0.01, ****p < 0.0001. Rotarod motor test (right panel) illustrating the latency to fall of accelerated rod in rotarod test at (**a**) age of 3 months (n = 3 WT, 5 TG), (**b**) age of 8 months (n = 7 WT, 7 TG) and (**c**) age of 13 months (n = 5 WT, 10 TG). Two-tailed student’s t-test: *p < 0.05. AMPH = amphetamine, m = meter, s = seconds.

In order to evaluate the effect of α-syn overexpression on motor activity in TG mice at different ages, we used two different motor tests, the open field test and the rotarod test. First, we examined the spontaneous and amphetamine-induced locomotor activity (5 mg/kg i.p.) of the TG mice compared to age-matched WT mice. At 3 months of age, the spontaneous locomotor activity was similar between WT and TG mice, and both groups responded to amphetamine with a significant increase in locomotor activity (Fig. 3a: left panel, 3 months, naive vs amphetamine: at WT p < 0.01, at TG p = 0.02). At the age of 8 and 13 months, WT mice significantly increased their locomotor activity upon amphetamine administration, however, TG mice failed to respond to stimulation of dopamine release by amphetamine with an increase in locomotor activity (Fig. 3a–c: left panel; 8 months, (naive vs amphetamine: WT p < 0.0001, TG n.s.; TG vs WT: naive n.s.; amphetamine p < 0.01) and at 13 month (naive vs amphetamine: WT p < 0.001, TG, n.s.; TG vs WT: naive n.s.; amphetamine p = 0.014)).

Next, we examined the performance of mice in the rotarod test, evaluating the latency to fall on an accelerating rotating rod as a measure of their gross motor skills. In this test, there was no difference in the latency to fall at ages 3 and 8 months, however, 13 months old TG mice had a significantly shorter latency to fall from the accelerated rod compared to age-matched WT mice (p = 0.046) (Fig. 3a–c: right panel). These findings confirmed the age-dependent behavioral impairment in the TG mice dividing them into asymptomatic (3 months old), symptomatic (8 months old) and severely affected (13 months old) stages of the disease based on their behavioral performance. The results were consistent with previous findings in this model^32^ and may be related to changes in dopamine synthesis and reuptake in the striatum as previously described^32^, and reflected in the decreased density of TH^**+**^ and DAT^**+**^ fibers.

### Blood–brain barrier leakage in TG mice

**Figure 4:**
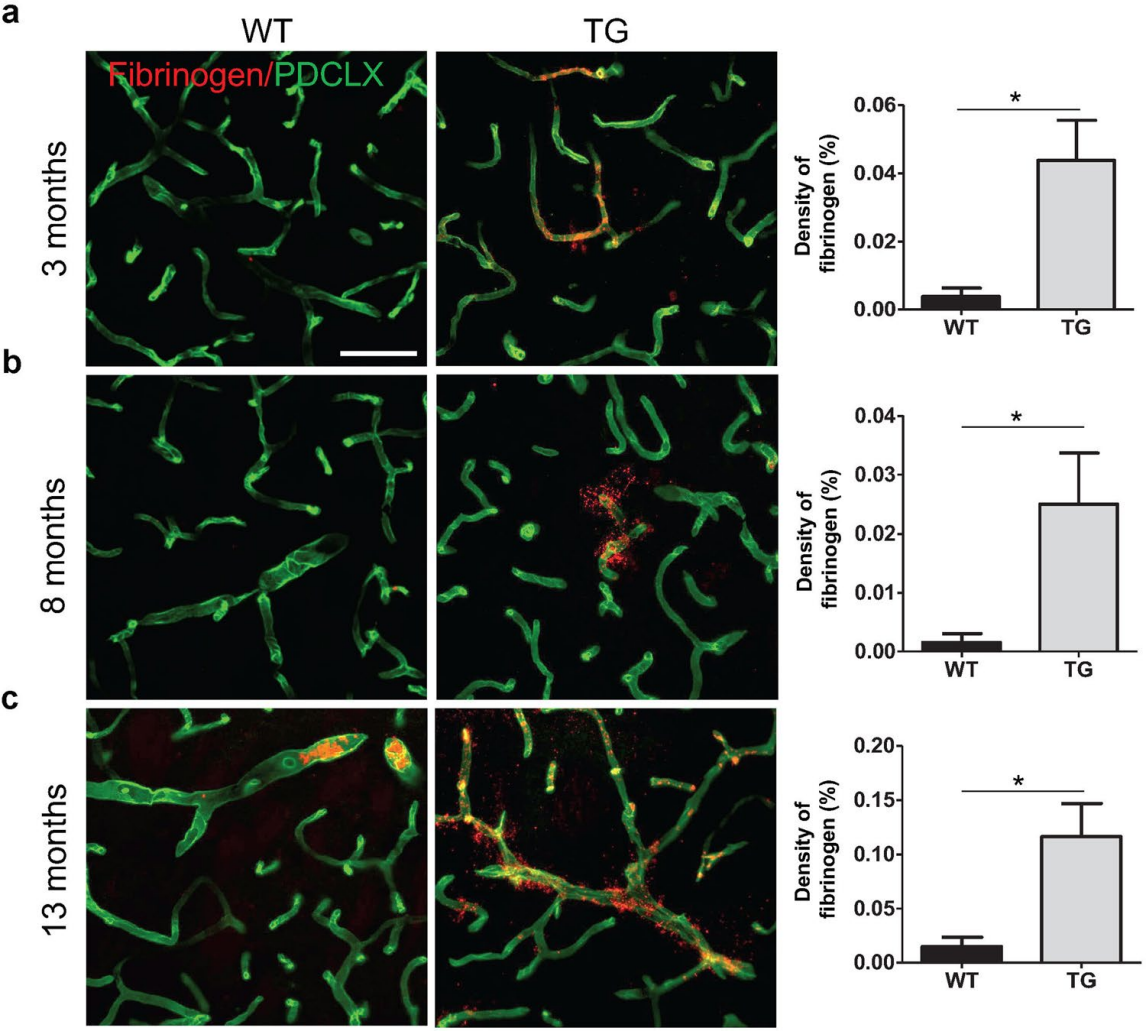
Increased fibrinogen leakage in the dorsal striatum of TG mice. Confocal images illustrating fibrinogen leakage in WT and TG mice and respective quantification showing extravascular fibrinogen density at (**a**) age of 3 months (n = 3 WT, 3 TG); (**b**) age of 8 months (n = 4 WT, 4 TG); and (**c**) age of 13 months (n = 4 WT, 4 TG). Two-tailed student’s t-test: *p-value < 0.05. Scale bar: 50 μm. PDCLX = Podocalyxin.

Evidence of BBB impairment has been described in PD patients^7,35^ and toxin-induced models of PD^23,24^ but it has not been investigated whether these pathological changes are also detected in PD models of α-syn overexpression. Next we asked whether TG mice have an impaired BBB integrity. We examined the presence of extravascular fibrinogen as an indicator of BBB leakage in the dorsal striatum, the area where the maximum reduction in the TH^**+**^ and DAT^**+**^ fiber density was observed. Extravascular fibrinogen accumulation was significantly increased in the striatum of TG mice compared to age-matched WT mice at 3, 8 and 13 months of age (3 months p = 0.02; 8 months p = 0.04; 13 months p = 0.02), indicating BBB leakage in α-syn overexpressing mice (Fig. 4a–c, and Supplementary Fig. S3).

### Age-dependent vascular changes but no alteration in pericyte density in TG mice

**Figure 5:**
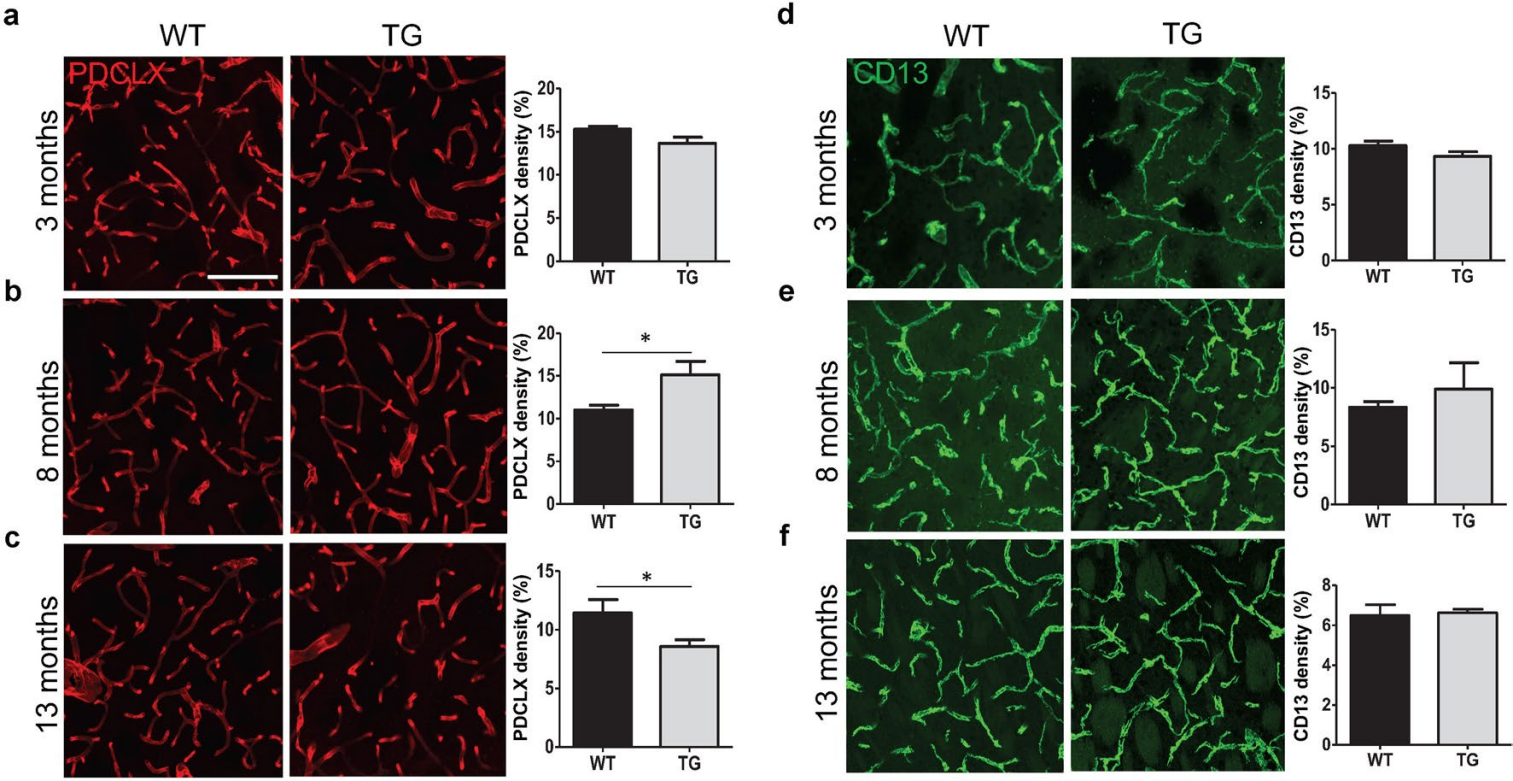
Alterations in striatal vessel and pericyte density in TG mice. (**a–c**) Confocal images illustrating PDCLX^+^ vessel density in WT and TG mice and quantification at (**a**) age of 3 months (n = 6 WT, 9 TG); (**b**) age of 8 months (n = 4 WT, 4 TG); and (**c**) age of 13 months (n = 7 WT, 8 TG). (**d–f**) Confocal images showing CD13^+^ pericyte density in WT and TG mice and quantification (**d**) at age of 3 months (n = 4 WT, 6 TG); (**e**) at age of 8 months (n = 4 WT, 4 TG); and (**f**) at age of 13 months (n = 7 WT, 5 TG). Two-tailed student’s t-test: *p-value < 0.05. Scale bar: 100 μm.

In the next step, we evaluated whether α-syn impacts on vessel density in TG mice. To do this, we analyzed the vessel density and particularly the pericyte density in the dorsal striatum where the maximum TH^+^ fiber loss had been observed. At 3 months of age, there was no detectable difference in PDCLX^+^ blood vessel density in TG mice compared to age-matched WT mice (p = 0.1) (Fig. 5a). In animals aged 8 months, we found an increased vessel density in TG mice compared to age-matched controls (p = 0.05) (Fig. 5b), whereas in 13 months old TG animals, the vessel density was significantly reduced compared to age-matched controls (p = 0.045) (Fig. 5c) indicating stage-dependent changes in vessel density in TG mice. The vascular changes observed in TG mice are likely related to an initial compensatory angiogenesis followed by vascular regression at later disease stages. WT mice showed a tendency of vascular regression from 3 to 8 months. The CD13^+^ pericyte density in TG mice was not affected at any of the investigated ages compared to age-matched WT mice (Fig. 5d–f).

**Figure 6:**
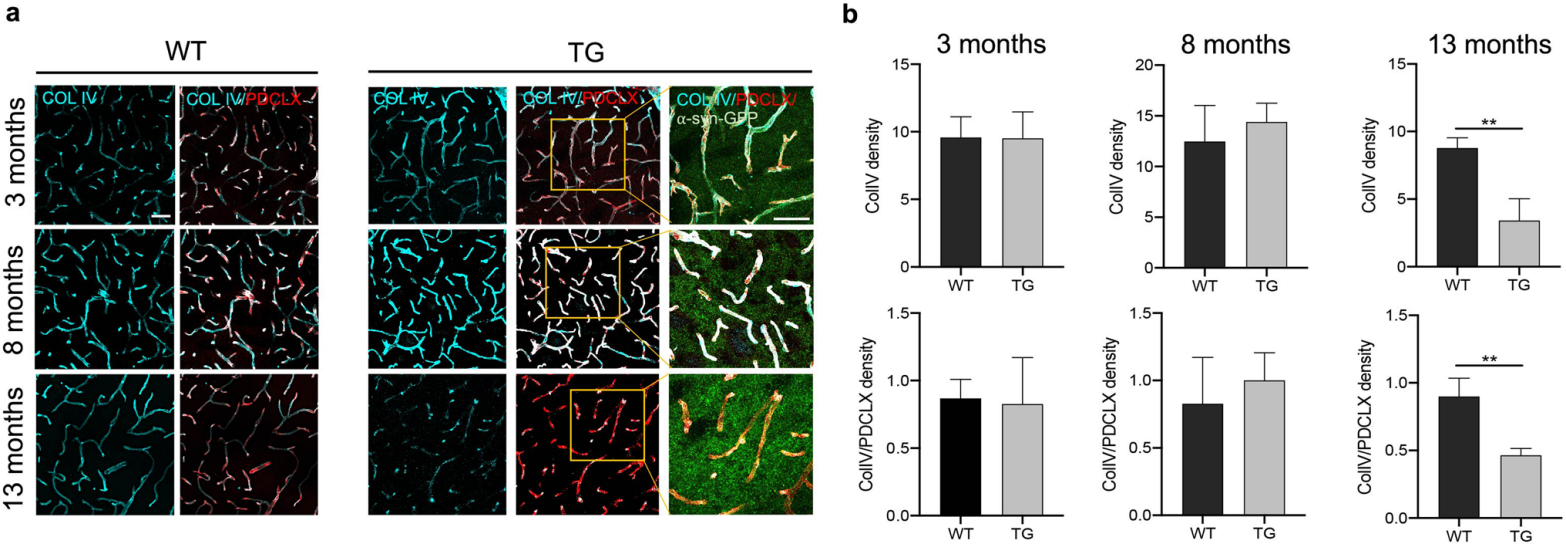
Reduction of Col IV in TG mice at late age. (**a**) Confocal images illustrating Col IV density and its proportion in relation to the PDCLX^+^ vessels in WT (left panel) and TG mice (right panel). (**b**) Quantification of Col IV density and its proportion in relation to the PDCLX^+^ vessels in WT and TG mice at age of 3 months (n = 3 WT, 3 TG); age of 8 months (n = 3 WT, 3 TG); and age of 13 months (n = 3 WT, 3TG). Two-tailed student’s t-test: *p-value < 0.05. Scale bar: 50 μm. PDCLX = Podocalyxin, Col IV = Collagen IV.

We also examined differences in the expression of Col IV, a marker for the basement membrane of capillaries, between WT and TG mice at 3 different ages. We found that the density of Col IV and its proportion in relation to the PDCLX^+^ vessels were significantly reduced in TG mice compared to WT mice at the age of 13 months (Col IV, p < 0.01; Col IV/ PDCLX, p < 0.01) (Fig. 6a,b).

### Early and sustained pericyte activation in TG mice

**Figure 7:**
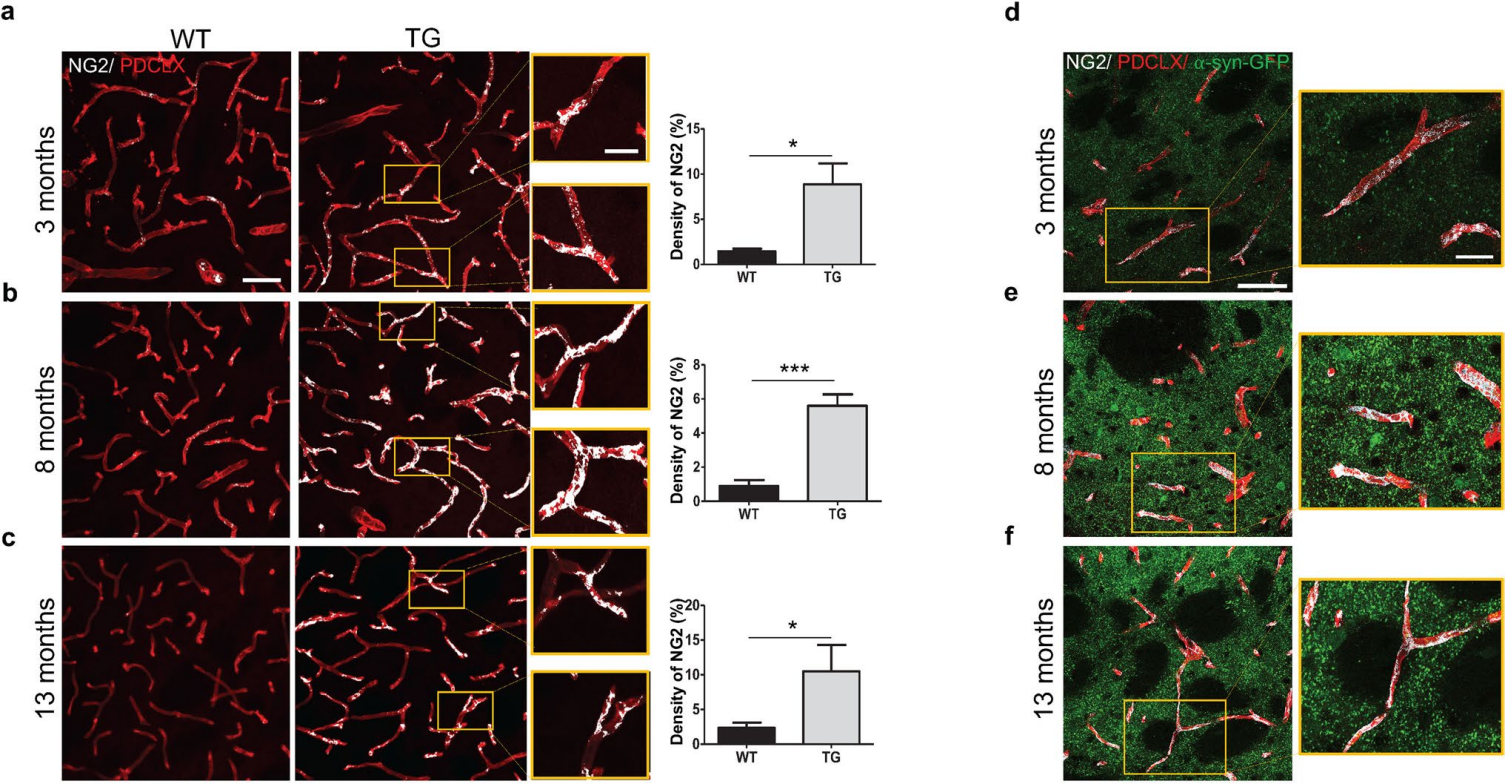
Activated NG2^+^ pericytes in the striatum of TG mice. Confocal images showing NG2^+^ pericyte coverage (grey) of PDCLX^+^ vessels (red) in WT and TG mice and quantification of NG2^+^ pericyte density at (**a**) age of 3 months (n = 3 WT, 3 TG); (**b**) age of 8 months (n = 4 WT, 3 TG); and (**c**) age of 13 months (n = 4 WT, 4 TG). Confocal images showing NG2^+^ pericyte coverage (grey) of PDCLX^+^ vessels with *α*-syn-GFP in TG mice at age of 3 (**d**), 8 (**e**) and 13 (**f**) months. Two-tailed student’s t-test: *p-value < 0.05, ***p-value < 0.001. Scale bar: large image (**a–f**) 50 μm; image box (**a–f**) 20 μm. PDCLX = Podocalyxin, NG2 = Neuron-glial antigen 2, α-syn-GFP = alpha-synuclein green fluorescent protein.

We have previously shown that pericytes are activated in a toxin-induced PD model^20^ and pathological pericyte activation is a feature of also other neurodegenerative disorders^12^. To further understand the vascular changes and in particular the pericyte response to α-syn, we investigated the proportions of blood vessels that are specifically covered by activated pericytes using labeling with NG2. NG2 is a marker that can identify perivascular pericytes undergoing activation^36^. The identification of NG2^+^ pericytes was based on their perivascular location and morphological criteria^37^. Interestingly, we observed an increase in the vessel coverage by NG2^+^ pericytes, which was significantly higher in the TG mice at all investigated ages when compared to age-matched WT mice (3 months, p = 0.03**;** 8 months p < 0.001; 13 months, p = 0.05) (Fig. 7a-f).

### α-syn-GFP and p-*a*-syn colocalize with endothelial cells but not with pericytes

**Figure 8:**
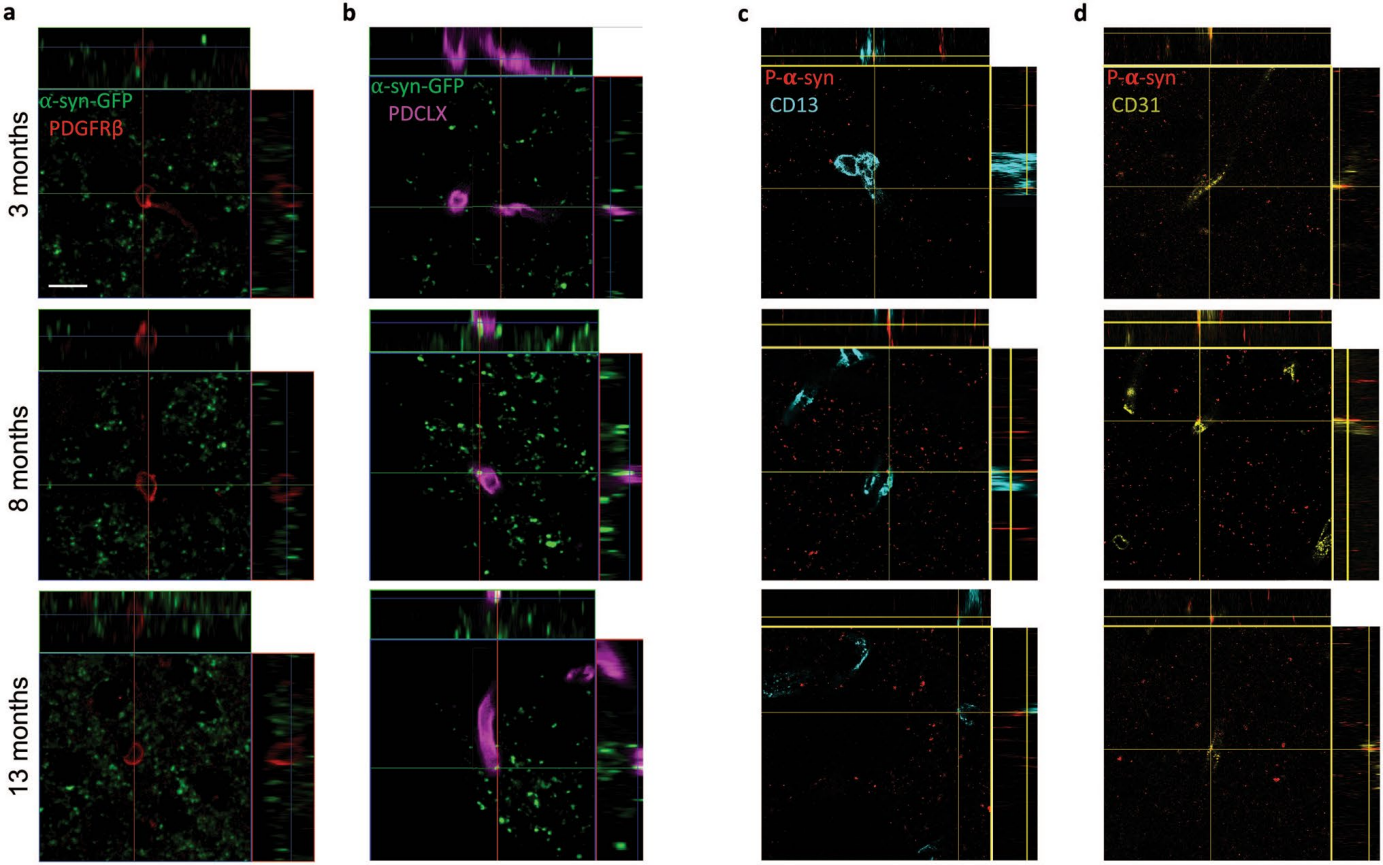
α-syn-GFP and pS129-α-syn inclusions are present in endothelial cells but not in pericytes. (**a**) Confocal pictures illustrating that α-syn-GFP does not co-localize with PDGFRβ^+^ pericytes (**b**) but shows co-localization with PDCLX^+^ blood vessels in the striatum of TG mice at age of 3, 8, and 13 months. (**c**) Confocal images showing pS129-α-syn does no colocalizate with CD13^+^ pericytes (**d**) but it colocalizes with CD31^+^ vessels in the striatum of TG mice at age of 3, 8 and 13 months. PDGFRβ = Platelet-derived growth factor receptor-beta, PDCLX = Podocalyxin, α-syn-GFP = alpha-synuclein green fluorescent protein, pS129-α-syn = phospho S129-alpha-synuclein. Scale bar: 10 µm.

To determine whether α-syn directly accumulates in cells forming capillaries, we examined the dorsal striatum for colocalization between α-syn-GFP or pS129 and PDCLX^+^ and CD31^+^ endothelial cells or PDGFRβ^+^ and CD13^+^ pericytes. Interestingly, we detected a colocalization between α-syn-GFP and pS129 with endothelial cells (Fig. 8b,d), but not with PDGFRβ^+^ or CD13^+^ pericytes (Fig. 8a,c).

## Discussion

**Figure 9:**
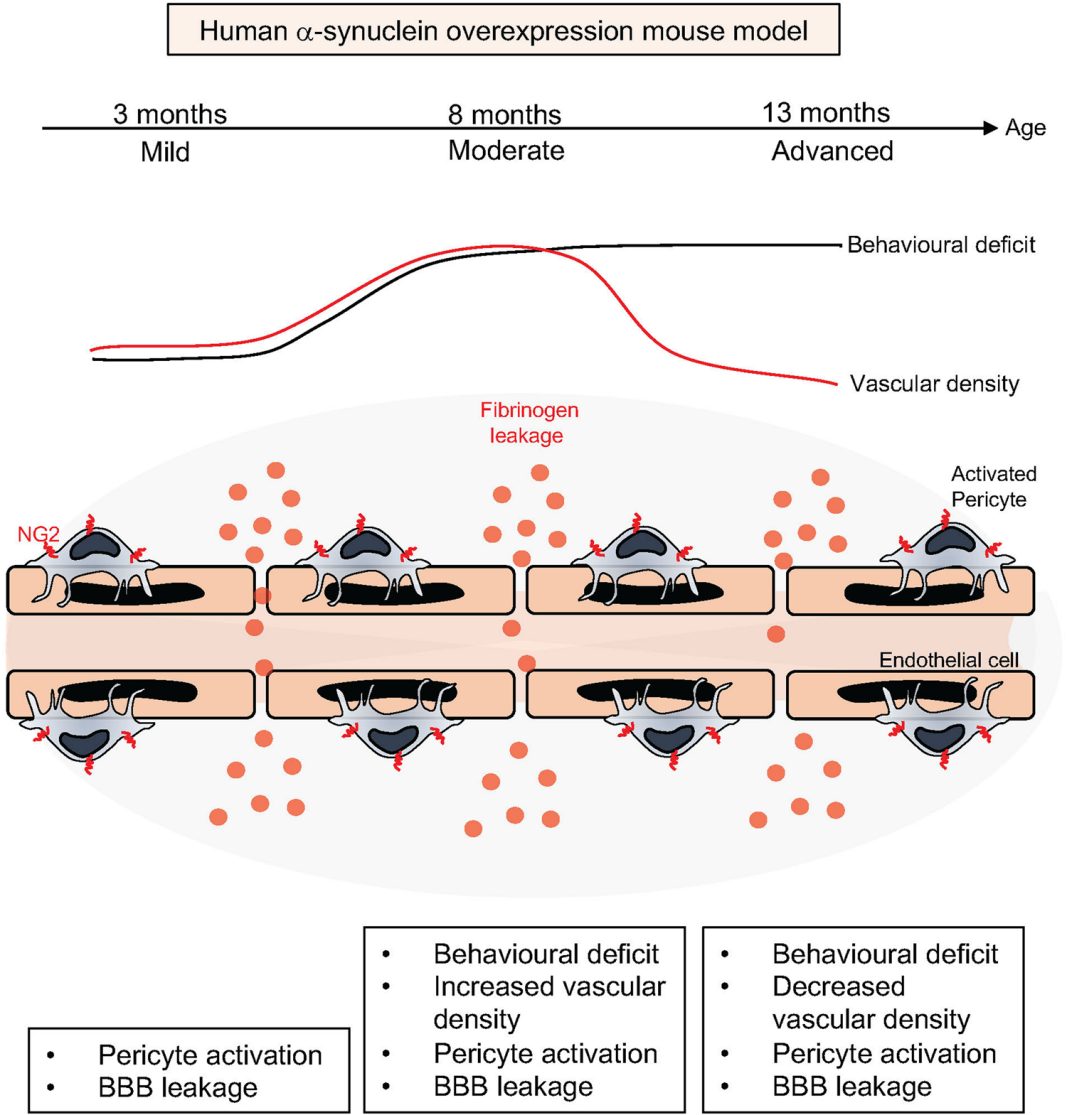
Summary of the key findings. Human α-syn overexpression mouse model displays early blood–brain barrier leakage, stage-dependent dynamic changes in vessel morphology and pathological activation of pericytes.

Here we examined pathological features of PD in a α-syn overexpression TG mouse model where human α-syn is overexpressed under the mouse α-syn promoter. We demonstrated an age-dependent increase in human α-syn and p-α-syn and show the occurrence of insoluble α-syn in this model as well as impaired striatal TH- and DAT- fiber density, progressive microglial activation and motor impairment. Importantly, we describe for the first time a compromised BBB integrity in this model that is detected already at early stages of the disease. This is associated with dynamic changes in vessel density and accompanied by a pathological activation of pericytes reflecting microvascular alterations. The findings are summarized in the graphical abstract (Fig. 9).

The TG mice used in this study exhibit a widespread expression of α-syn including the DA neurons of the SNpc and ventral tegmental area, the neocortex, as well as other brain regions^32^. The model has previously been characterized and shown to have a progressive increase of α-syn aggregation with age and a gradual reduction of striatal dopamine release^32^.

Here we have confirmed that with increasing age, TG mice exhibited α-syn and p-α-syn accumulation resulting in insoluble α-syn that is associated with a significant reduction in striatal DAT- and TH-fibers and a progressive motor impairment, confirming the progressive nature of the model^32^. The observed behavioural impairment could not be related to a significant cell death of the TH neurons in the SNpc by the end of study, but rather reflects defects in dopamine synthesis, release and synaptic transmission^32,38^. Even though this might be perceived as an imbalance between striatal and nigral pathology, the neuropathological sequence in some of the transgenic models overexpressing α-syn requires several month to develop a clinical phenotype and yet often presents with a lack of overt loss of DA neurons in the SNpc^38,39^. A large body of evidence indicates that neurodegenerative alterations start in nerve terminals, such as terminal swellings and loss of nerve terminals^40^. It has also been shown that α-syn overexpression first impacts on vesicle organization at the DA synapse seen as deficits in dopamine release in the dorsal striatum long before cell loss is seen in the SNpc^39^. A similar neuropathological pattern has been observed in models using AAV-driven overexpression of α-syn^41^. Those progressive degenerative changes seen over time support the idea that the α-syn-induced pathology first affects the axons and terminals and later progresses to involve also the cell bodies, strikingly similar to the retrograde progression of neurodegeneration seen in human PD. The striatal changes are reflected in defects in dopamine neurotransmission^32^ and consistent with our present findings showing a significant reduction in striatal DAT and TH fibers in TG mice reflecting the impact of α-syn on TH-expression^42^ and DAT activity^43^.

The widespread α-syn pathology in PD is not only toxic to the nigrostriatal system. Interestingly, we found that TG mice develop an early accumulation of extravascular plasma fibrinogen indicating BBB leakage. Increased extravasation of erythrocytes, perivascular hemosiderin deposits and leakage of serum proteins such as haemoglobin and fibrinogen has been described in post-mortem PD brain sections indicative of BBB leakage^7,35^. These findings in post-mortem studies are supported by the previous animal work in MPTP and 6-OHDA PD mouse models^23,24^. In contrast to our study, post-mortem analysis and toxin-induced PD models render interpretation of temporal aspects of BBB dysfunction more difficult and toxin-induced PD models do not fully resemble the progressive α-syn aggregation characteristic of PD. However, both models share the occurrence of neuroinflammation that may play a role in the observed microvascular pathology. We noted that extravascular fibrinogen was already detectable in TG mice at 3 months, indicating that BBB changes occur early and even before behavioural impairment manifests in these TG mice.

We found the density of microvessels to be dynamically altered in TG mice. In contrast to the age-related reduction in vessel density described in the literature^44,45^ that was also evident in WT mice, the vascular density in TG mice first significantly increased at 8 months before it was reduced at 13 months. This increase in vessel density at the moderate stage of the disease in TG mice is likely the result of a compensatory angiogenesis. Formation of new blood vessels requires angiogenesis, which in the adult brain is only stimulated under pathological conditions^46^. Evidence for angiogenesis and increased vessel numbers has been reported in toxin-induced animal models of PD^23,47^ and in human PD^4,6^. Angiogenesis is coupled to immature leaky vessels, consistent with our observation of BBB leakage. Neuroinflammation, as also observed in this model, may contribute to the impaired BBB^48^, however, we also noted an increase in activated NG2 labelled pericytes^19^ already at the pre-symptomatic stage at 3 months. Pericytes can respond to pathological stimuli with activation^49,50^, which results in changes in marker expression, such as NG2, an indicator of an angiogenic response that indicates vessel remodelling^18^. It is conceivable that both, angiogenesis and BBB leakage are mediated by activated pericytes. Interestingly, we detected α-syn and p-α-syn in endothelial cells, but not in pericytes. Also, even though there was a change in vessel density, the pericyte coverage was unaffected, suggesting that α-syn accumulation does not lead to pericyte loss and that not pericyte loss per se, but rather the activation of the cells may be the cause of the observed BBB changes.

Activated pericytes not only change their marker expression, but also alter their signalling^12,51,52^ which impacts on neighbouring cells^49,51^. Interestingly, α-syn has been shown to require the presence of pericytes to induce hyperpermeability in endothelial cells^51^*. *In vitro*,* α-syn exposure induced secretion of high amounts of pro-inflammatory mediators in pericytes^51^ which may contribute to the observed disruption of the BBB even in the absence of microglia^53,54^. It is conceivable that α-syn-induced dysregulation of BBB integrity depends on pericytes also in vivo. Together with their role in angiogenesis, this would place pericytes in a key position between α-syn and BBB leakage and at the same time, identify them as target cells.

Pericyte dysfunction has been shown to contribute to neurodegeneration in a variety of brain disorders^55^. Pathological pericyte activation has been described in other neurodegenerative diseases characterized by abnormal protein aggregates such as Huntingtons disease, where pericyte activation even precedes neuronal loss^12^. We have recently described a pathological activation of pericytes in the striatum in a partial 6-OHDA lesion mouse model of PD^20^. When mice were treated with PDGF-BB, this not only induced neurorestoration and behavioural recovery^20,56^, but also normalized the number of activated pericytes^20^. We have shown that PDGF-BB can change the secretome of pericytes towards a trophic factor pattern in vitro^57^. PDGF-BB has already been investigated in a phase I/IIa clinical trial in PD. Consistent with observations in animal models^20,56^, the study demonstrated a dose-dependent significant improvement in DAT as measured by positron emission tomography (PET)-Scan whereas the signal declined in placebo patients^58^. We have suggested that the disease-modifying mechanism of PDGF-BB is mediated by normalization of the abnormal vasculature in PD^20^, and possible changes in the secretome of pericytes^57^. Future studies are now needed to investigate the in vivo secretome of pericytes and endothelial cells in α-syn PD models in order to confirm this hypothesis. Addressing the secretome of pericytes could potentially prevent secondary BBB disruption and point to new avenues for therapeutic approaches targeting the neurovascular unit in PD.

When we examined TG mice at the severe stage (13 months), we identified a reduction in vessel density compared to age-matched WT mice consistent with vessel regression. Interestingly, also vascular regression such as reduction in vascular length, vascular branching points and diameter^7,8^ as well as the formation of string vessels have been observed in human post-mortem PD brain sections^35^. The exact mechanisms underlying the vascular remodelling in PD are still sparsely studied. It is conceivable that angiogenesis is a compensatory response to injury, attempting to restore circulation and providing necessary oxygenation and nutrients from the blood. This response might be initiated by pericytes. At later stages, this attempt for vascular remodelling likely fails due to progressive neuropathological changes resulting in vascular degeneration.

In summary, we describe evidence for multiple microvascular changes that can be modeled in an α-syn overexpression PD mouse model. Our findings support that the microvasculature in PD undergoes both, an angiogenic and pruning vascular response and add information on the temporal sequence of vascular alterations. We show that occurrence of BBB leakage and neovascularization is an early event in disease progression and vascular degeneration a sign of late stage disease in this model. Vascular alterations and BBB disruption are now increasingly recognized as a common denominator of many neurodegenerative disorders^3^ and the resulting accumulation of serum proteins or toxins in the parenchyma likely further exacerbate pathology. Therefore, mechanisms underlying the BBB breakdown may help to develop interventions to protect the BBB, prevent vascular degeneration or enhance vascular remodelling. This model provides a powerful tool to investigate aggravating and disease-modifying factors in PD in a temporal sequence that might guide the development of new treatments.

## Supplementary Information

Supplementary Information.
