# Supplementary material for: Human α-synuclein overexpression in a mouse model of Parkinson’s disease leads to vascular pathology, blood brain barrier leakage and pericyte activation
Source: Sci Rep. 2021 Jan 13;11:1120. doi: 10.1038/s41598-020-80889-8 (PMC7806665; doi:10.1038/s41598-020-80889-8)
Supplement: Supplementary file 1 — Supplementary Information. [file 41598_2020_80889_MOESM1_ESM.pdf]

Human α-synuclein overexpression in a mouse model of Parkinson's disease leads to vascular pathology, blood brain barrier leakage and pericyte activation

Osama Elabi[1], Abderahim Gaceb[1], Robert Carlsson[1], Thomas Padel[1], Rana Soylu-Kucharz[2], Irene Cortijo[1], Wen Li[3], Jia-Yi Li[3,4] and Gesine Paul[1,5*].

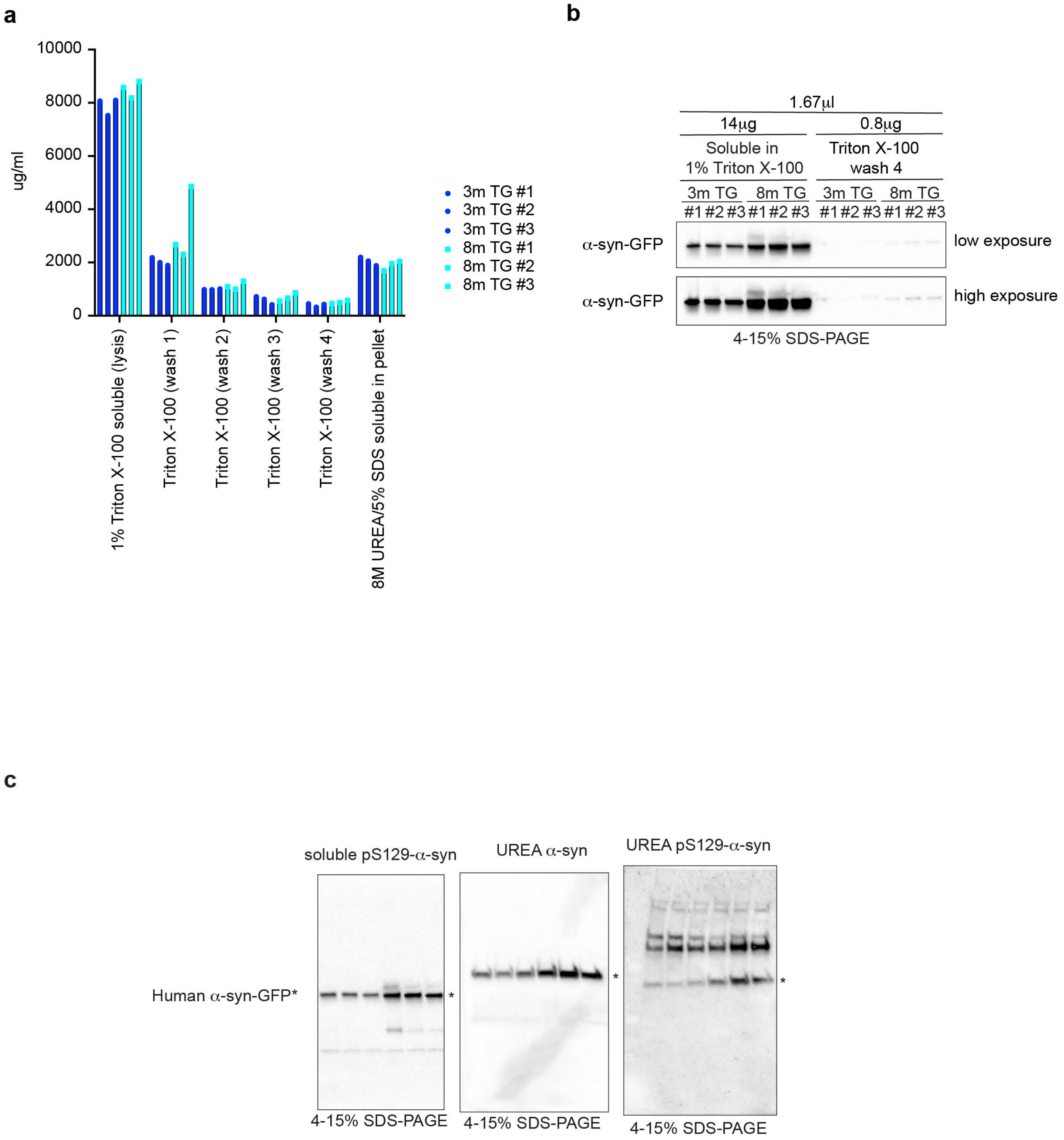
a
10000
8000
6000
4000
2000
0
ug/ml
3m TG #1
3m TG #2
3m TG #3
8m TG #1
8m TG #2
8m TG #3
1% Triton X-100 soluble (lysis)
Triton X-100 (wash 1)
Triton X-100 (wash 2)
Triton X-100 (wash 3)
Triton X-100 (wash 4)
8M UREA/5% SDS soluble in pellet
b
1.67μl
14μg
0.8μg
Soluble in 1% Triton X-100
Triton X-100 wash 4
3m TG
8m TG
3m TG
8m TG
#1 #2 #3 #1 #2 #3 #1 #2 #3 #1 #2 #3
α-syn-GFP
low exposure
α-syn-GFP
high exposure
4-15% SDS-PAGE
c
soluble pS129-α-syn
UREA α-syn
UREA pS129-α-syn
Human α-syn-GFP*
*
4-15% SDS-PAGE
4-15% SDS-PAGE
4-15% SDS-PAGE

Supplementary figure 1

Verification of fractionation method

(a) Bicinchoninic Acid (BCA) measurement of total protein concentration in equal volumes of 1% Triton X-100 lysates, washes #1-4, and UREA/SDS fractions. (b) Specific detection of α-syn in equal volumes of 1% Triton X-100 lysates and wash #4 supernatant. (c) Whole images of the western blots in Figure 1c and 1d. Asterix (*) indicates human α-syn-GFP at 50KDa.

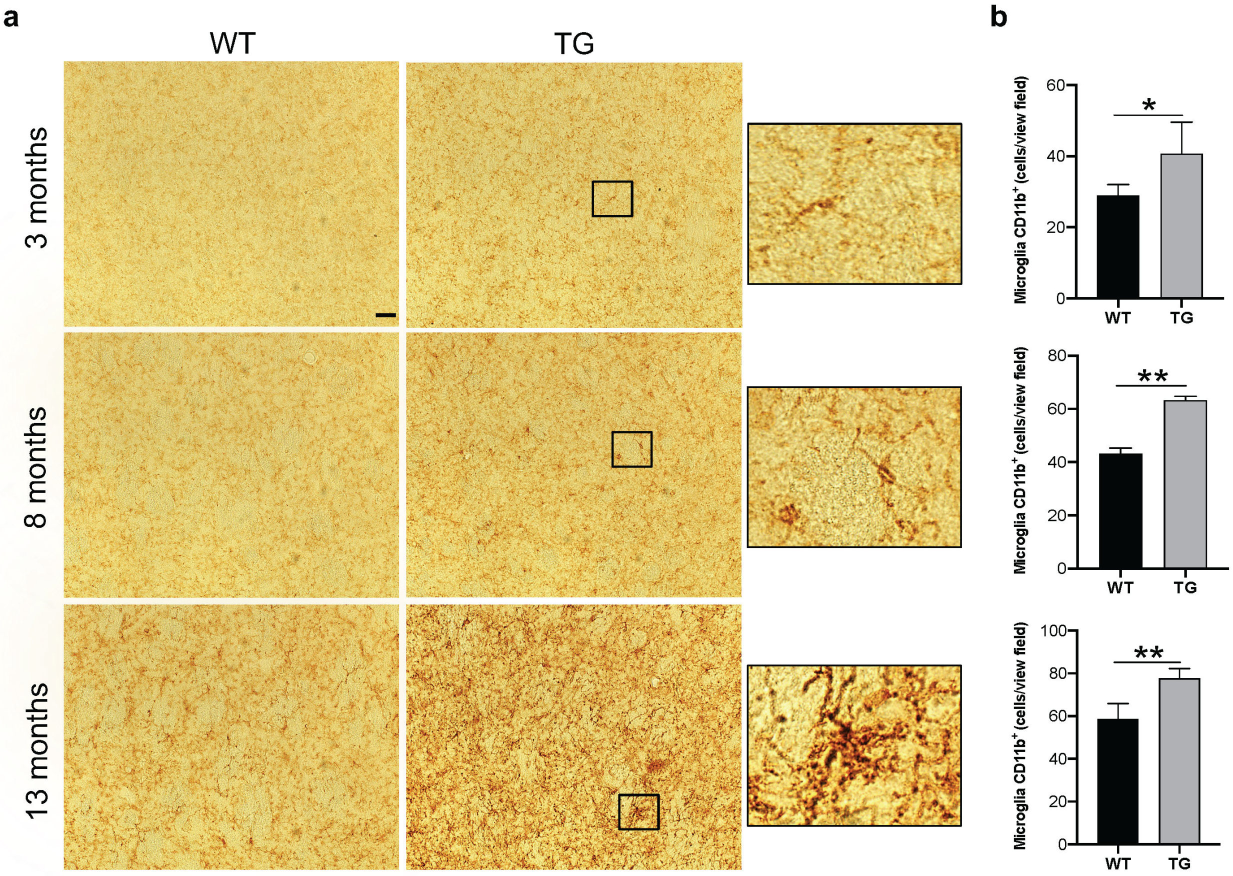
a
WT
TG
3 months
8 months
13 months
b
Microglia CD11b+ (cells/view field)
WT
TG
*
**
**

Supplementary figure 2

$CD11b^+$ microglia in the dorsolateral striatum of WT and TG mice. (a) Images showing $CD11b^+$ microglia in WT and TG mice at 3, 8, and 13 months of age. Zoomed images illustrating $CD11b^+$ microglia morphology. (b) Quantification of $CD11b^+$ cell number per view field at 3 months (n= 4 WT, 4 TG); 8 months (n= 3 WT, 2 TG); and 13 months of age (n= 5 WT, 4 TG). Two-tailed student's t-test: $^*p<0.05$, $p^{**}<0.01$. Scale bar: 20 μm.

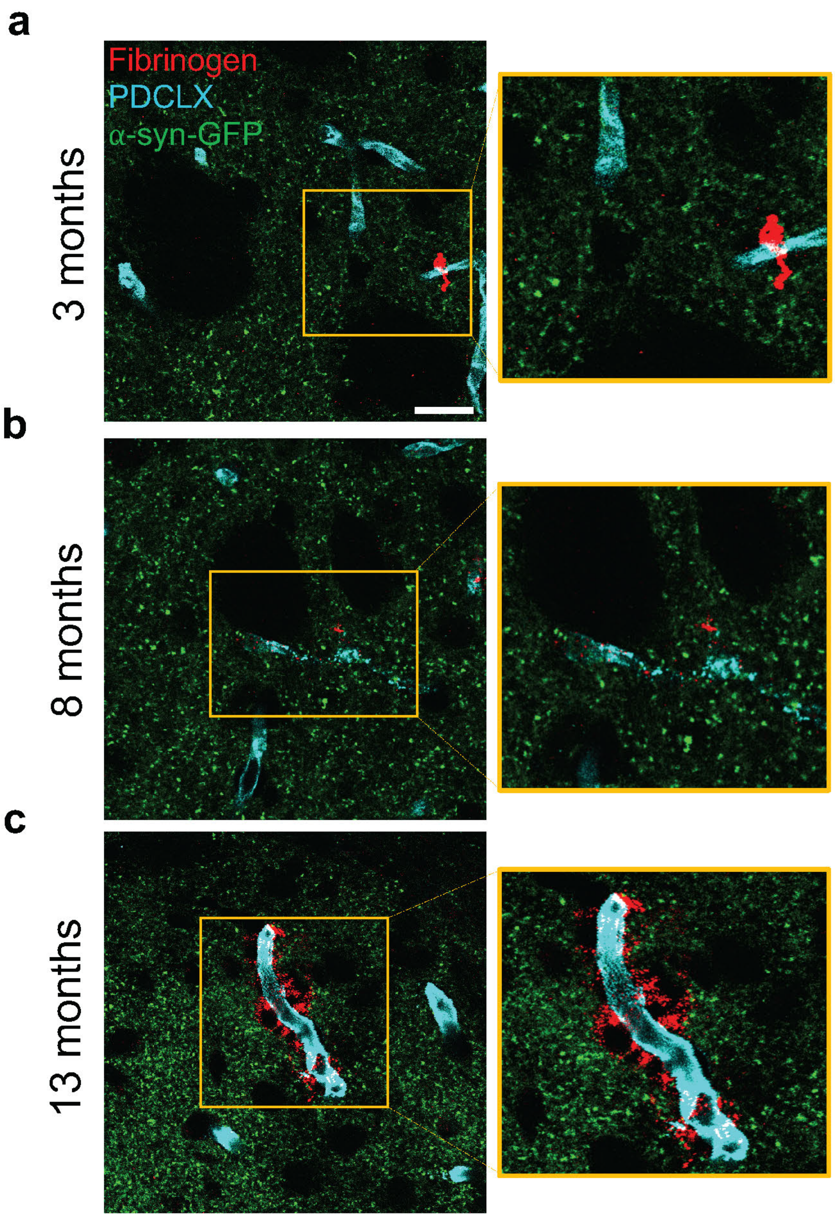
a
b
c
3 months
8 months
13 months
Fibrinogen
PDCLX
α-syn-GFP

Supplementary figure 3

Extravascular leakage of fibrinogen in relation to α-syn-GFP. Confocal images showing extravascular leakage of fibrinogen (red), PDCLX+ vessels (cyan) with α-syn-GFP (green) in the striatum of TG mice at (a) age of 3 months, (b) age of 8 months, and (c) age of 13 months. PDCLX= Podocalyxin, α-syn-GFP = alpha-synuclein green fluorescent protein. Scale bar: 20μm.

Supplementary material and methods

One brain hemisphere per mouse was kept ice cold and cut to small pieces with a scalpel blade. 1 ml of ice cold lysis buffer (1% Triton X-100 in 100 mM NaCl, 50 mM Tris-HCl, 1 mM EGTA, 10 mM
MgCl2, pH 7.2 with x1 Halt protease (#78430) and x1 Halt phosphatase inhibitors (#1862495)) incubated 5min at room temperature (rt) and then spun 16100g at +4°C for 1 min. The supernatant was kept as the soluble fraction and the insoluble fraction was resuspended in lysis buffer to wash the insoluble fraction. This was repeated 3 times to generate a four time's washed, insoluble pellet. The soluble fractions of the washes were saved as fractions: wash #1-4. The insoluble pellet was solubilised in UREA-buffer (8M Urea, 5% SDS, 10mM EGTA in dH_2O) and sonicated three times in a sonicator (Qsonica) for 10s at 30% amplitude. Protein concentrations were measured with BCA kit using standard procedures (Thermo scientific, Pierce BCA protein kit, cat no #23225). Protein lysates were resuspended in 4x laemmli sample buffer to a final added concentration of 50 mM Tris-HCl pH 6.8, 2% SDS, 10% glycerol, 12.5 mM EDTA, 0.02 % bromophenol blue, and supplemented with 0.1M Dithiothreitol (DTT). The samples were then incubated at 95°C for five minutes prior to loading maximum 14μg per well. BioRad Mini-protean TGX gels were run 200V for 30 min. The proteins were then transferred from the gels to nitrocellulose membranes (Trans-Blot Turbo Transfer Pack, #1704159, BioRad). The membranes were blocked 1h at room temperature in 5% weight/volume skim milk in 1x PBS pH 7.4, containing 0.1 % Tween-20 (PBS-T). Antibodies used for the detection of α-syn were; human α-syn-GFP (BD #610786, 1:1000) or for pS129-α-syn-GFP (Abcam #ab51253, 1:1000). The primary antibodies were incubated 1h at rt with agitation. Following x3 5min 10ml PBS-

T washes, secondary rabbit-anti-mouse-HRP (1:5000, P0260, Dako) or goat-anti-rabbit (1:5000, ab6721, Abcam) were added in 5% milk in PBS-T and incubated for 1h at rt with agitation. The blots were then washed x3 for 5 min in PBS-T. Clarity, or Clarity max (Biorad) were used as a substrate for chemiluminiscent detection of HRP in a MP Chemidoc. Images were acquired in Imagelab version 5.2.1 and quantified in Fiji-ImageJ.
